# Reply to Çınar, A. The Potential Role of Organ-to-Liver Ratios in Immune-Related FDG PET Imaging. Comment on “Panikar et al. Exploratory FDG PET/CT Imaging in mCRPC Patients Treated with Sipuleucel-T ± IL-7: A Phase II Trial Subanalysis. *Int. J. Mol. Sci.* 2026, *27*, 5129”

**DOI:** 10.3390/ijms27188197

**Published:** 2026-09-15

**Authors:** Sandeep Surendra Panikar, Dhruv Bansal, John Crandall, Hari Raman, Joel Picus, Joseph E. Ippolito, Daniel L. J. Thorek, Richard L. Wahl, Russell K. Pachynski

**Affiliations:** 1 Department of Medicine, Washington University School of Medicine, St. Louis, MO 63110, USA; panikar@wustl.edu (S.S.P.);; 2 Alvin J. Siteman Cancer Center, Barnes-Jewish Hospital, Washington University School of Medicine, St. Louis, MO 63110, USA; 3 Departments of Radiology and Radiation Oncology, Washington University School of Medicine, St. Louis, MO 63110, USA; 4 Department of Biomedical Engineering, Washington University, St. Louis, MO 63130, USA

We sincerely thank Dr. Alev Çınar and colleagues for their thoughtful and constructive comments [1] regarding our recently published article, “Exploratory FDG PET/CT Imaging in mCRPC Patients Treated with Sipuleucel-T ± IL-7: A Phase II Trial Subanalysis” [2]. We are grateful for their positive assessment of our work and for highlighting important considerations regarding quantitative PET biomarkers for assessing systemic immune activation during cancer immunotherapy. We fully agree that harmonized PET quantification is essential for improving the reproducibility and inter-study comparability of quantitative imaging biomarkers. We applied a standardized FDG PET method for absolute quantitation. This absolute quantitation using the QIBA profile approach has proven repeatability [3].

However, it can be cumbersome and normalization of splenic and bone marrow FDG uptake to hepatic activity using the spleen-to-liver ratio (SLR) and bone marrow-to-liver ratio (BLR) may provide a practical approach to partially account for inter-scan and inter-patient variability while preserving clinically relevant information.

As noted by Dr. Çınar and others [4,5,6,7], accumulating evidence supports the prognostic and biologic relevance of these normalized immune-organ biomarkers in patients receiving immunotherapy; however, these associations should not be interpreted as evidence that SLR or BLR can predict treatment response. Much of the existing evidence for these normalized immune-organ biomarkers has been generated in melanoma and other immunotherapy settings, and their clinical applicability in metastatic castration-resistant prostate cancer (mCRPC) remains uncertain. These findings therefore provide a rationale for investigation rather than establishing the clinical validity of SLR or BLR in mCRPC [4,5,8]. We do caution that methods normalizing spleen to the liver are potentially limited by (1) the compounded variabilities inherent in measuring tracer uptake in both the reference tissue and the liver, (2) assumptions that the liver is free of disease and immunological activity, and (3) the time sensitivity of FDG uptake over time in the liver (declining with time) vs. the potential rise in FDG uptake in the spleen over time. Thus, liver-to-spleen or liver-to-marrow ratios may be somewhat more time-sensitive (post-injection) than absolute organ SUV measurements. BLR introduces additional sources of variability, including heterogeneous marrow distribution, differences in hematopoietic activity, systemic inflammation, treatment-related changes in marrow metabolism, and the presence and extent of skeletal metastatic disease [4,5]. Thus, both SLR and BLR may be sensitive to post-injection timing and other biological factors, and these considerations should be addressed through standardized acquisition protocols and prospective validation.

We concur that quantitation of splenic and marrow activity levels by FDG PET is important as we work to non-invasively interrogate the function of the immune system on a whole-body level with PET imaging. Our study was designed as an exploratory imaging subanalysis of a multicenter Phase II clinical trial evaluating sipuleucel-T with or without recombinant human interleukin-7 (CYT107) in patients with metastatic castration-resistant prostate cancer [2,9]. Because participation in the imaging substudy was optional, only three patients underwent serial FDG PET/CT imaging. Consequently, the principal objective was to characterize longitudinal metabolic changes associated with treatment rather than to establish or validate quantitative imaging biomarkers. Within this exploratory framework, we focused on absolute changes in FDG uptake over time. Importantly, our observation of increased splenic FDG uptake following treatment raises the hypothesis that FDG PET/CT may capture treatment-induced systemic immune activation in addition to alterations in tumor metabolism. Given the exploratory design and the inclusion of only three patients, this observation should be considered hypothesis-generating rather than evidence of a validated imaging biomarker. We believe these preliminary findings provide an important foundation for future investigations of immune-response imaging in prostate cancer. We agree that incorporation of normalized immune-organ biomarkers, including SLR and BLR, into larger prospective immunotherapy studies has the potential to strengthen interpretation of immune-related metabolic changes, improve quantitative standardization across imaging centers, and facilitate the development of robust and reproducible PET biomarkers for treatment response assessment. Importantly, although existing studies suggest prognostic and biologic associations, the predictive value of SLR and BLR for immunotherapy response remains unproven and requires prospective validation [4,5,8].

We appreciate Dr. Çınar and colleagues for emphasizing these important future directions. Prospective studies incorporating standardized PET acquisition, clinical endpoints, and immunologic correlates will be essential to determine the clinical utility of SLR and BLR in mCRPC.

## Data Availability

No new data were created or analyzed in this study. Data sharing is not applicable to this article.
